# Association between Soluble α-Klotho Protein and Metabolic Syndrome in the Adult Population

**DOI:** 10.3390/biom12010070

**Published:** 2022-01-04

**Authors:** Yung-Wen Cheng, Chun-Chi Hung, Wen-Hui Fang, Wei-Liang Chen

**Affiliations:** 1 National Defense Medical Center, Department of Family and Community Medicine, Division of Family Medi-cine, School of Medicine, Tri-Service General Hospital, Taipei 114, Taiwan; cyw0451@gmail.com (Y.-W.C.); rumaf.fang@gmail.com (W.-H.F.); 2 National Defense Medical Center, Department of Orthopaedic Surgery, School of Medicine, Tri-Service General Hospital, Taipei 114, Taiwan; raffi10110126@gmail.com; 3 National Defense Medical Center, Department of Family and Community Medicine, Division of Geriatric Medicine, School of Medicine, Tri-Service General Hospital, Taipei 114, Taiwan; 4 National Defense Medical Center, Department of Biochemistry, Taipei 114, Taiwan

**Keywords:** Klotho, FGF23, metabolic syndrome

## Abstract

Klotho protein is an anti-aging protein and plays multiple roles in ion-regulation, anti-oxidative stress, and energy metabolism through various pathways. Metabolic syndrome is a combination of multiple conditions that compose of multiple risk factors of cardiovascular disease and type 2 diabetes. Gene regulation and protein expression are discovered associated with metabolic syndrome. We aimed to figure out the correlation between Klotho protein and metabolic syndrome in generally healthy adults. A cross-sectional study of 9976 respondents ≥ 18 years old from the US National Health and Nutrition Examination Survey (2007–2012) by utilizing their soluble Klotho protein concentrations. Multivariate linear regression models were used to analyze the effect of soluble Klotho protein on the prevalence of metabolic syndrome. Soluble Klotho protein concentration was inversely correlated with the presence of metabolic syndromes (*p* = 0.013) and numbers of components that met the definition of metabolic syndrome (*p* < 0.05). The concentration of Soluble Klotho protein was negatively associated with abdominal obesity and high triglyceride (TG) in the adjusted model (*p* < 0.05). Soluble Klotho protein is correlated with changing metabolic syndrome components in adults, especially central obesity and high TG levels. Despite conventional function as co-factor with fibroblast growth factor-23 (FGF23) that regulates phosphate and vitamin D homeostasis, FGF23-independent soluble Klotho protein may act on multiple signal pathways in different organs and tissue in roles of anti-aging and protection from metabolic syndrome.

## 1. Introduction

The klotho gene was originally identified as a gene that inherited a syndrome resembling human aging, encoding Klotho protein and expressing mainly in the kidneys, brain, parathyroid glands [1,2,3,4,5]. Klotho proteins are essential components of endocrine fibroblast growth factor (FGF) receptor complexes, as they are required for the high-affinity binding of FGF19, FGF21, and FGF23 to their cognate FGF receptors (FGFRs). There are three subfamilies of Klotho: α-Klotho, β-Klotho, and γ-Klotho. α-Klotho activates FGF23, and β-Klotho activates FGF19 and FGF21 [6]. The word “Klotho” generally means α-Klotho. Klotho can exist in a membrane-bound form, a soluble, circulating, or a secreted form, potentially functioning as an endocrine factor [3]. Classically, Klotho protein is a co-factor of FGF23, which regulates phosphorus and vitamin D homeostasis [2,3,7,8,9,10]. It has been postulated that the extracellular domain of Klotho protein is clipped on the cell surface and secreted into the bloodstream, in the form of soluble Klotho protein, acts variously on target tissues and plays roles in hormonal function, and governs multiple metabolic processes in mammals [2,3,5,7,8,10].

Metabolic syndrome is the combination of several metabolic abnormalities that are highly associated with increased risks for developing diabetes, stroke, and cardiovascular disease (CVD). The components of metabolic syndrome include central obesity (waist circumference over 35 inches or 88 cm of women and over 40 inches or 102 cm of men), insulin resistance (fasting serum glucose over 100 mg/dL or under-treatment of diabetes), hypertension (SBP > 130 mmHg or DBP > 85 mmHg or under medical treatment of hypertension), high triglycerides (TG > 150), and low high-density lipoprotein (HDL) cholesterol (HDL < 40 in men or HDL < 50 in women). The National Cholesterol Education Program’s Adult Treatment Panel III (NCEP ATP III) defines metabolic syndrome as having three or more of the above traits [11].

With age increasing, the reduction of Klotho protein leads to several diseases, and health conditions increase with age. Multiple cardiovascular risk factors, including hypertension, diabetes, obesity, and hyperlipidemia, were discovered in a rat model [12]. On the other hand, the prevalence of metabolic syndrome increases with age, degree of obesity, and propensity to type 2 diabetes. Since the Klotho protein is known as an anti-aging protein, and it has much to do with energy metabolism. Our study aimed to figure the correlation between Klotho concentration and metabolic syndrome.

## 2. Materials and Methods

### 2.1. Study Design and Participant Selection

Study participants were adults aged 20 and over enrolled in the National Health and Nutrition Examination Survey (NHANES), which is a nationally representative, complex sampling survey that combines interviews and the results of physical examinations. From 2007–2008, 2009–2010, and 2011–2012 study cycles, 11,128 respondents were screened, and those with missing data were excluded. Finally, 9976 participants were recruited. All data were acquired from the NHANES website. The use of the NHANES data was approved by the National Center for Health Statistics (NCHS) Research Ethics Review Board, and all participants provided written informed consent. Participants were divided into 2 groups based on the presence or absence of metabolic syndrome. Definition of metabolic syndrome is according to the NCEP ATP III panels, in the presence of 3 or more of the following criteria: waist circumferences (>102 cm in men or >88 cm in women), hyperglycemia (fasting serum glucose over 100mg/dL or under-treatment of diabetes), hypertension (SBP ≥ 130 mmHg or DBP ≥ 85 mmHg or under medical treatment of hypertension), high triglycerides (TG > 150 mg/dL), and low HDL-cholesterol (HDL < 40 mg/dL in men or HDL < 50 mg/dL in women) [11].

### 2.2. Measurement of Klotho Protein

The study measure soluble α-Klotho protein, which is a pleiotropic protein that acts on multiple targets. Pristine serum samples were obtained by the laboratory at NHANES, National Center for Health Statistics, Centers for Disease Control and Prevention. Samples were received on dry ice and stored at −80 degrees Celsius until they were provided for analysis. Analysis was performed by a commercial ELISA kit produced by IBL International, Japan. There is no specific cut-point of Klotho that can indicate the biological age of a human. Samples with high and very high Klotho concentrations used different dilutions. The expected value and the obtained value showed good linearity in the measurement range (R^2^ = 0.998 and 0.997, respectively). The intra-assay precision exhibited a coefficient of variation (CV) of 3.2% and 3.9% for 2 recombinant samples and 2.3% and 3.3% for 2 human samples. The inter-assay CV of 2.8% and 3.5%; 3.8% and 3.4%, respectively. The same sample will be analyzed twice, and the result will be the average of the 2 values. Quality control samples with low and high concentrations were also analyzed in duplicate in each ELISA kit. If the value of the quality control sample is not within the 2SD range, the entire analysis results will be rejected, and the analyses repeated. The obtained assay sensitivity was 6 pg/mL. The reference value of α-Klotho levels ranged from 285.8 to 1638.6 pg/mL (mean: 698.0 pg/mL) [13].

### 2.3. Biochemical Profiles

Standard biochemical profiles were sampled from the 2007–2008 NHANES database. Including aspartate aminotransferase (AST), creatinine (Cr), cholesterol, and glucose levels, the 21 analytes constituted the routine biochemistry profile. The serum specimen was store under −30 °C conditions until testing by National Center for Environmental Health. The DxC800 system uses an enzymatic rate method to determine AST, Jaffe rate method for creatinine, oxygen rate method to measure glucose in serum. University of Minnesota, Minneapolis, MN conducted the tests of blood lipid levels, including HDL-cholesterol and triglyceride. Roche Modular P chemistry analyzer was used to determine HDL-cholesterol and TG by enzymatic method with endpoint reaction. The linear range of the method: 0–120 mg/dL for HDL-cholesterol. The analytical measurement range for TG was 0–1000 mg/dL.

### 2.4. Other Covariates

Covariates included: age, gender, race/ethnicity, body mass index obtained from the demographic variable, and body measures examination. Self-reported diagnoses were identified by the question “has a doctor or other health professional ever told you that you had (coronary heart disease and angina/angina pectoris)” (yes/no). Participants with drugs used for high blood pressure and diabetes were identified from the data of prescription medication questionnaire. Smoking data were provided from the questionnaire on cigarette use.

### 2.5. Statistical Analysis

Continuous variables were expressed as mean ± SD. Moreover, categories variables were expressed as numbers and percentages. Statistical significance was analyzed with t-tests or Chi-square tests using SPSS 18.0 (SPSS Inc., Chicago, IL, USA) for Windows. Multi-variant linear regression analysis was used for Klotho protein concentrations and continuous variables for each component (waist circumference, blood pressure, and glucose, and lipid profiles). Model 1 was unadjusted, whereas Model 2 was adjusted for age, gender, race-ethnicity, BMI, serum AST, serum creatinine, coronary artery disease, angina/angina pectoris, and smoking. *p* < 0.05 indicates a statistically significant difference.

## 3. Results

### 3.1. Characteristics of the Study Participants

A total of 9976 adults were included in the study and separated into the metabolic syndrome group (*N* = 3906) and the non-metabolic syndrome group (*N* = 6070). There were significant differences between both groups with respect to baseline characteristics (Table 1). The mean age of the metabolic syndrome group at baseline was 58.93 years (SD 10.84) and 56.64 years (SD 10.83) of the non-metabolic syndrome group. With respect to the distribution by gender, 48.1% and 50.3% were men in the metabolic syndrome group and non-metabolic syndrome group.

### 3.2. Association between the Klotho Protein and the Metabolic Syndrome Components

Table 2 provides Klotho protein concentrations and the presence of metabolic syndrome. In the adjusted model, Klotho protein concentration was inversely associated with the presence of metabolic syndrome (*p* = 0.013). Table 3 demonstrates the correlation between the Klotho protein concentration and metabolic syndrome components. In the adjusted model, the Klotho protein concentration inversely correlated with the number of metabolic syndrome components (3: *p* < 0.001 and 4–5: *p* = 0.002). In statistical analysis of each component, Klotho protein concentrations were inversely correlated with abdominal obesity (*p* < 0.001) and high TG (*p* < 0.001). A positive correlation was noted between Klotho protein concentration and high glucose. (*p* = 0.002).

## 4. Discussion

This is the first study to demonstrate the association between metabolic syndrome and soluble Klotho protein concentration in generally healthy adults. According to our result, there is a significant negative association between the prevalence of metabolic syndrome and Klotho protein concentration. Among the metabolic syndrome components, negative associations were disclosed for abdominal obesity, as well as high triglycerides.

### 4.1. The Basic Biological Role of Klotho Protein

With increasing age, the reduction of Klotho protein in vivo, which leads to several diseases and health conditions, increased with age. In a rat model with multiple cardiovascular risk factors, including hypertension, diabetes, obesity, and hyperlipidemia, in vivo Klotho gene delivery can ameliorate vascular endothelial dysfunction [12]. The contribution of Klotho leads to the shrinkage of endothelial function companies with a decrease of nitric oxide (NO) and enhancement of vasoconstriction. The calcification of vessel walls and reduction of vascular elasticity may be an important reason for elderly hypertensive incidence [14]. Klotho expression and production were suppressed in spontaneously hypertensive rats and the renal protected effects [15]. α-Klotho, β-Klotho, FGF21, and FGF23 proteins are expressed in cardiomyocytes. Comparing subjects classified by cardiovascular risks, cardiac atria biopsy samples disclosed reducing expression of cardiac Klotho, and an elevated expression of cardiac FGFs was associated with higher CV cardiovascular risks [16].

### 4.2. Klotho Protein and Glucose and Lipid Metabolism

Aging is considered associated with impaired glucose tolerance and type 2 diabetes mellitus. The Klotho mutant mouse is a novel non-obese animal model for decreased insulin secretion and production [17]. α-Klotho knock-out mice experience atrophy in many metabolic organs, including adipose tissue and liver [18]. In addition, the knock-out of the Klotho gene in leptin-deficient mice reduces obesity and increases insulin sensitivity [10]. Obesity is considered one of the main risk factors of metabolic syndrome. Although there were no changes in food intake, body weight, or blood glucose levels, α-Klotho-treated mice had reduced obesity, increased lean mass, and increased energy expenditure [19]. CSF α-Klotho’s prominent role in energy balance was demonstrated by strong inverse correlations with body weight and an ability to increase energy expenditure [20]. Soluble Klotho improves hepatic glucolipid homeostasis to ameliorate diabetic phenotypes and lipid accumulation in the mice model with type 2 diabetes mellitus in comparing wild-type, soluble Klotho heterozygous, and soluble Klotho transgenic group [21]. Histological examination suggests that Klotho mice possess less energy storage than wild-type mice concerning glycogen in the liver and lipid in brown adipose tissue [22]. The positive association between high blood glucose and Klotho was found significant in our study. In the review of the literature, one hypothesis suggested that in subjected overnutrition, the Klotho protein may induce insulin resistance to oppose the life-shortening consequences of lipotoxicity and lipoapoptosis [23]. Klotho interferes with insulin-mediated phosphorylation, blocks insulin-stimulated uptake of glucose, and decreases malonyl CoA, thereby promoting fatty acid oxidation, reducing intracellular lipid content, and raising the apoptotic threshold, and extending the life of the cells [24]. The balance of Klotho regulation of insulin resistance or insulin sensitivity is remained unclear, while the signaling mechanism responsible for α-Klotho-mediated changes in lipid-related gene expression is still unknown. At present, further research is needed to explore the role of Klotho protein in different terminal organs and the relationship between glucose and lipid metabolism.

### 4.3. Klotho Protein and Intracellular Signaling

Previous data indicate that soluble Klotho levels are not affected in type 2 diabetes patients with and without the macrovascular disease [25]. Furthermore, hyperglycemia does not affect renal Klotho production. Soluble Klotho levels are not associated with deteriorated kidney function in chronic kidney disease patients and have poor predictive value for all-cause mortality after a 2-years follow-up [25]. A role for α-Klotho in the regulation of muscle progenitor cell mitochondrial function and implicate α-Klotho declines as a driver of impaired muscle regeneration with age [26]. Gene and protein expression analyses show that α-Klotho is abundantly expressed in rodents and humans in the kidney and the choroid plexus of the brain, and to a lesser extent in areas such as the parathyroid gland, thyroid gland, pancreas, and sex organs [7]. Wnt signaling inhibits lipid accumulation, promotes lysosomal function and intracellular cholesterol trafficking and regulates blood pressure, and enhances insulin signal [27,28]. α-Klotho binds to different types of Wnt ligands to suppress the downstream signaling transduction, and that the α-Klotho knock-out increases Wnt signaling in mice [4,29,30]. Klotho protein regulates the activity of multiple glycoproteins on the cell surface, including ion channels and growth factor receptors such as insulin/insulin-like growth factor-1(IGF-1) receptors, which results in oxidative stress suppression [2,7]. Soluble Klotho could inhibit the PI3K/AKT/mTORC1 signaling to upregulate peroxisome proliferator-activated receptor α (PPARα) expression by directly interacting with type 1 insulin-like growth factor (IGF1) receptor in high fat diet-fed type 2 diabetes mellitus mice [10].

### 4.4. Klotho Protein and FGF Family

Transmembrane Klotho protein is a co-factor with FGF23, which regulates phosphorus and vitamin D metabolism. Some studies supported the hypothesis that aging is due to dysregulated phosphate metabolism, which results in massive calcification in most tissues and organs [9]. A cross-sectional cohort study measured circulated FGF23 and discovered the correlation between FGF23 and body composition and lipid profile. FGF23 levels were higher in subjects with the metabolic syndrome compared with those without and associated with an increased risk of having the metabolic syndrome [31]. Serum FGF23 levels are elevated in obese individuals, especially those with abdominal obesity. The presence of abdominal obesity and the increase in serum FGF23 levels in men and postmenopausal women [32]. FGF receptor (FGFR) could be directly activated by FGF23 in the absence of Klotho [33]. Klotho-independent FGF23 binding to FGFR4 and stimulates the PLCγ/calcineurin/NFAT cascade, which is associated with inducing expression of the inflammatory cytokines in hepatic and cardiac cells [34,35]. Independent to the FGF family, the expression of Klotho protein in the cardiovascular (CV) system and immune cells involves modulating the inflammation, including inhibition of NF-κB activity, suppression of Wnt biological activity, and regulation of the intrinsic generation of reactive oxygen species (ROS) [29,30,33,36,37]. On the other hand, Klotho displays the β-glucuronidase and sialidase activity and modifies the function of transporters in the kidney and intestine [38]. Since FGF23 may be an indicator of the risk of metabolic syndrome, Klotho protein implies its role against metabolic diseases. Currently, no evidence of a specific Klotho protein receptor has been found. Further in vivo data are needed to support the physiological contributions of soluble Klotho protein independent to FGF23 in the mechanism of metabolic syndrome.

### 4.5. Limitation

As with the majority of studies, the design of the current study is subject to limitations. First, this is a cross-sectional study, it could not access causality between Klotho and metabolic syndrome. Although NHANES sets to recruit a randomized representative sample of the U.S. population, those who participated in the research visit may differ in subtle ways from those who did not, which may affect the results of this study. Second, the value of Klotho protein in this study is measurable soluble α-Klotho protein. Serum Klotho exhibits circadian variations, and the blood samples were not obtained at a certain time. The function of different subtypes of Klotho protein, transmembrane Klotho protein, plays roles in different signal pathways, which in the mice study is more important in development than soluble Klotho. However, our study could not measure the effects of transmembrane Klotho. Moreover, limited information on the percentage of soluble Klotho could represent the total Klotho levels. Third, the effect of FGF family and vitamin D deficiency were considered associated with the metabolic syndrome. Limited data are provided in the NHANES study cycles could be obtained [39]. Thus, we are unable to provide the results with correction for FGF and Vitamin D concentration of respondents.

## 5. Conclusions

Our study showed the inverse correlation between Klotho protein and the presence of some components of the metabolic syndrome (abdominal obesity and high TG in a generally healthy adult population. The prevalence of metabolic syndromes increases with age. Klotho protein is known to be an anti-aging protein, first discovered in the renal tubule and found to influence multiple organs and tissues. The biological roles of Klotho proteins include anti-oxidative stress, suppression of the signal of inflammatory processes, regulation the phosphate and vitamin- D metabolism, and energy metabolism. The result of this study strengthens the potential of protective roles of Klotho protein and the possibility of independence to FGF families in metabolic syndrome and further therapeutic strategy.

## Figures and Tables

**Table 1:** Characteristics of participants with and without metabolic syndrome.

Variables	*N* = 9976		
	Metabolic Syndrome *N* = 3906	Non-Metabolic Syndrome *N* = 6070	*p* Value
**Continuous variables**, **mean(SD)**			
Age (years)	58.93(10.84)	56.64(10.83)	<0.001
Body mass index (kg/m^2^)	32.40(6.29)	27.65(5.85)	<0.001
Serum creatinine (mg/dL)	0.94(0.45)	0.91(0.49)	0.012
Serum AST (U/L)	27.20(17.31)	25.99(14.95)	<0.001
Klotho (pg/mL)	848.35(292.92)	871.54(311.68)	<0.001
**Metabolic syndrome component, mean(SD)**			
SBP (mmHg)	133.63(19.25)	123.63(17.39)	<0.001
DBP (mmHg)	73.52(13.54)	71.26(11.49)	<0.001
Waist circumference (cm)	109.38(13.98)	96.45(14.03)	<0.001
Serum TG (mg/dL)	239.42(186.63)	120.11(73.57)	<0.001
Serum HDL (mg/dL)	43.67(12.00)	58.91(16.05)	<0.001
Serum glucose (mg/dL)	125.34(55.94)	96.73(27.19)	<0.001
**Categorical variables, n (%)**			
Gender			0.035
Male	1880(48.1)	3053(50.3)	
Female	2026(51.9)	3017(49.7)	
Race			<0.001
Mexican American	724(18.5)	778(12.8)	
Other Hispanic	458(11.7)	618(10.2)	
Non-Hispanic White	1829(46.8)	2731(45.0)	
Non-Hispanic Black	645(16.5)	1335(22.0)	
Other Race—Including Multi-Racial	250(6.4)	608(10.0)	
Past history			
Coronary heart disease	258(6.6)	233(3.8)	<0.001
Angina/angina pectoris	180(4.6)	129(2.1)	<0.001
Smoke	2017(51.6)	2886(47.5)	0.001

SBP, Systolic blood pressure; DBP, Diastolic blood pressure TG, triglycerides; HDL, High-density lipoprotein; AST, aspartate aminotransferase; SD, standard deviation.

**Table 2:** Relation between klotho and metabolic syndrome.

	Metabolic Syndrome	
	β (95% CI)	*p* Value
Model 1	−23.127 (−35.396, −10.857)	<0.001
Model 2	−16.652 (−29.790, −3.514)	0.013

Model 1 = unadjusted. Model 2 = Model 1 + age, gender, race-ethnicity, body mass index (BMI), serum aspartate aminotransferase (AST), serum creatinine, coronary artery disease, angina/angina pectoris, and smoking.

**Table 3:** Regression coefficients of the presence and number of metabolic syndrome components with Klotho.

	Klotho			
Number of Metabolic Syndrome Components	Model 1		Model 2	
	β (95% CI)	*p* Value	β (95% CI)	*p* Value
Presence of metabolic syndrome				
1	−33.097 (−54.357, −11.837)	0.002	−29.141 (−50.554, −7.727)	0.008
2	−38.584 (−59.529, −17.639)	<0.001	−29.792 (−51.554, −7.865)	0.008
3	−54.985 (−76.466, −33.503)	<0.001	−45.598 (−68.782, −22.414)	<0.001
4–5	−48.195 (−70.745, −25.645)	<0.001	−38.364 (−63.182, −13.546)	0.002
*p* for trend	<0.001		<0.001	
**Each metabolic syndrome component**				
Abdominal obesity	−19.508 (−31.946, −7.070)	0.002	−31.545 (−47.901, −15.188)	<0.001
High blood pressure	−15.179 (−27.794, −2.564)	0.018	−4.571 (−17.367,8.225)	0.484
High triglycerides	−41.340 (−53.436, −29.245)	<0.001	−34.401 (−46.640, −22.162)	<0.001
Low HDL cholesterol	−3.799 (−16.715,9.117)	0.564	−4.371 (−17.534, 8.792)	0.515
High glucose	6.494 (−5.686,18.673)	0.296	19.627 (7.185, 32.068)	0.002

HDL, High-density lipoprotein. Model 1 = unadjusted. Model 2 = Model 1 + age, gender, race-ethnicity, Body mass index (BMI), serum aspartate aminotransferase (AST), serum creatinine, coronary artery disease, angina/angina pectoris, and smoking.

## Data Availability

NHANES data are publicly available at https://wwwn.cdc.gov/nchs/nhanes/Default.aspx (accessed on 5 July 2021).
